# Is exponential stability achievable in singular perturbed delayed systems with time-varying parameters? A comprehensive analysis

**DOI:** 10.1016/j.heliyon.2024.e27424

**Published:** 2024-03-09

**Authors:** Ran Chen, Min Ouyang, Jinju Zhang, Fatemeh Masoudinia

**Affiliations:** a School of Electronic Science and Engineering, Hunan University of Information Technology, Changsha, 410151, China; b Wuling Power Corporation LTD., Changsha, 410004, China; c School of Computer & Communication Engineering, Changsha University of Science & Technology, Changsha 410004, China; d Department of Electrical Engineering, Sofyan Branch, Islamic Azad University, Sofyan, Iran

**Keywords:** Singular perturbed systems, Exponential stability, Delayed systems, Time-varying parameters, Multiple time scales

## Abstract

The present article conducts an investigation into the phenomenon of exponential stability within singular perturbed delayed systems, incorporating time-varying parameters. Singularly perturbed systems serve as essential tools in modeling intricate systems characterized by multiple time scales, wherein one subsystem exhibits significantly faster evolution than the others. The presence of small delays introduces complexities, influencing both state derivatives and delays, further accentuating the intricacies of the system. Drawing upon the principles of singular perturbation theory, the article introduces a novel approach to analyzing the stability of these complex systems, eschewing the conventional assumption of exponential stability in the fast subsystem. Within the scope of this study, we propose a rigorous stability analysis, utilizing Linear Matrix Inequality (LMI) methods, while considering time-varying parameters that exert substantial influence on the system's dynamics. The proposed methodology enables the exploration of system stability beyond conventional assumptions, imparting valuable insights into the behavior of singular perturbed delayed systems amidst varying conditions. Through extensive numerical simulations, the effectiveness and robustness of the approach are validated, illuminating the stability properties of these intricate systems. Comparative studies with existing techniques, which assume exponential stability in the fast subsystem, demonstrate the distinct advantages and uniqueness of the presented approach. The findings underscore the significance of accounting for time-varying parameters in achieving a comprehensive understanding of the exponential stability inherent in singular perturbed delayed systems. This research makes substantial contributions to the field of system stability analysis, particularly in the context of singular perturbed delayed systems featuring time-varying parameters. The originality of our approach lies in introducing a comprehensive analysis framework that overcomes the limitations of existing methodologies. By integrating a novel stability analysis method based on Linear Matrix Inequalities (LMIs), we offer a fresh perspective on achieving exponential stability in such complex systems. Significantly, our work addresses a critical gap in current literature by challenging the assumption of exponential stability in the fast subsystem, a key feature of singularly perturbed systems. Through a meticulous examination of time-varying parameters, we unveil their profound impact on system dynamics, thus enriching the understanding of stability behaviors. The potential real-world applications of our findings span diverse fields, ranging from engineering to mathematical modeling. Performance metrics are a key focal point of our research. Numerical simulations employing our proposed LMIs serve as a robust benchmark, demonstrating the superior stability achieved in comparison to existing methods. This performance-driven evaluation ensures the practical applicability and reliability of our analysis approach across various scenarios.

## 1 Introduction

### 1.1 Motivation

Singularly perturbed systems have demonstrated their efficacy as potent mathematical tools for effectively capturing intricate interactions among subsystems characterized by distinct temporal characteristics [[1], [2], [3]]. These versatile systems find wide-ranging applications across various domains, encompassing chemical reactions, electrical circuits, ecological models, and neural networks [[4], [5], [6]]. Gaining a comprehensive understanding of the stability properties of such systems holds paramount importance in ensuring their robustness and dependability in real-world applications [[7], [8], [9]]. Moreover, the incorporation of time-varying parameters provides an avenue for achieving a more precise and faithful representation of the inherent complexities in these systems, thereby affording a deeper insight into their dynamic behavior under varying conditions [[10], [11], [12]]. The presence of multiple time scales in singularly perturbed systems gives rise to unique dynamics, where certain subsystems evolve significantly faster than others [[13], [14], [15]]. Such intricate interactions are pervasive and inherent in numerous natural and engineered systems [[16], [17], [18], [19]]. The behavior of these systems is notably sensitive to initial conditions and external influences, leading to complex and often unpredictable responses [[20], [21], [22], [23], [24]]. Consequently, characterizing the stability properties of singular perturbed systems becomes a central concern in their analysis and application [[25], [26], [27], [28], [29]].

Robustness and reliability are critical attributes that singularly perturbed systems must exhibit to ensure their suitability for real-world implementation [[30], [31], [32]]. A thorough examination of their stability under different operating conditions is, therefore, imperative [33,34]. Traditional stability analysis methods often rely on the assumption of exponential stability in the fast subsystem, simplifying the analysis but limiting the insights into system behavior [35,36]. To address this limitation, this study proposes a novel approach to stability analysis, one that abstains from assuming exponential stability in the fast subsystem, thus paving the way for a more comprehensive exploration of stability properties [[37], [38], [39]]. Furthermore, the incorporation of time-varying parameters in the analysis is paramount for capturing the intricate nature of real-world systems, where parameters may not remain constant over time [[40], [41], [42]]. In many practical applications, parameters are subject to variations, uncertainties, or external disturbances [[43], [44], [45]]. Hence, incorporating time-varying parameters in the stability analysis enriches the understanding of the system's behavior under diverse scenarios, enhancing its practical relevance [[46], [47], [48], [49], [50]].

In light of the above considerations, this research contributes to the broader understanding of singularly perturbed systems and their stability properties. By unveiling new perspectives on stability analysis and embracing the influence of time-varying parameters, this study aims to extend the knowledge base of these complex systems. The proposed approach and findings hold significant implications for diverse scientific and engineering disciplines, enabling more accurate predictions and ensuring the robustness of singularly perturbed systems in real-world applications.

**Table:** 

Nomenclatures	
$E_{\varepsilon }$	The diagonal matrix dependence on the constant value ε
$I_{n}$ and $I_{m}$	Correspond to identical n×n and m×m identity matrices
h(t)	The discrete delay
r(t)	The distributed delay
εh(t)	Discrete piecewise continuous delay
εr(t)	Distributed piecewise continuous delay
$h_{0}$	The upper bounds of the discrete delay
$r_{0}$	the upper bounds of the distributed delay
$z_{t}$	The system's states
${\dot{z}}_{t}$	The derivatives of the system's states
φ	The initial function
W	The Sobolev space
δ	The converging rate

### 1.2 Literature review

The stability properties of singular perturbed systems and the consequences of delayed dynamics have been the subject of extensive investigation in numerous studies [[51], [52], [53], [54], [55]]. Traditional methodologies employed in these studies have commonly assumed exponential stability in the fast subsystem, yielding valuable insights across diverse contexts [[56], [57], [58]]. Notably, the emergence of LMI methods has revolutionized the analysis of singularly perturbed systems, offering efficient techniques for stability analysis and providing a deeper understanding of their dynamic behavior [[59], [60], [61]]. Despite the significant advancements in stability analysis facilitated by LMI methods, a notable limitation persists in the existing literature [62,63]. Specifically, the consideration of time-varying parameters, a crucial aspect of many practical systems, has been frequently overlooked. Real-world systems often exhibit variations or uncertainties in parameters over time due to environmental changes, operational conditions, or external influences [[64], [65], [66]]. The omission of time-varying parameters in the analysis can result in an incomplete representation of system dynamics, potentially leading to inaccurate or overly simplified stability assessments [67,68].

Recognizing the importance of accounting for time-varying parameters in stability analysis, this study aims to address this research gap and presents a novel approach that embraces the influence of such parameters on singularly perturbed systems. By incorporating time-varying parameters within the LMI-based stability analysis, a more comprehensive and accurate characterization of system behavior under varying conditions is achieved. This approach seeks to broaden the understanding of the stability properties of singularly perturbed systems and unlock their full potential for real-world applications. In conclusion, while traditional approaches have laid a solid foundation for stability analysis in singularly perturbed systems, this study endeavors to extend the scope of analysis by considering time-varying parameters, thereby enriching insights into system dynamics. By leveraging the power of LMI methods and accounting for the influence of time-varying parameters, this research aims to advance the state-of-the-art in stability analysis and contribute to the practical relevance and applicability of singularly perturbed systems in diverse scientific and engineering domains.

Study [69], investigates the stability analysis of singularly perturbed systems with time-varying delays. The authors propose a novel approach to analyze the stability properties of such systems by considering the impact of time-varying delays on system behavior. The research explores how delays can influence the stability of singularly perturbed systems and offers valuable insights into the dynamics of complex systems with varying delays. In the article [70], the authors present a stability analysis for a specific class of singularly perturbed systems with time-varying delay. They develop a novel analytical approach to investigate the stability behavior of such systems under different conditions. The research contributes to the understanding of how time-varying delays can affect the overall stability and behavior of singularly perturbed systems, which is crucial for various engineering and control applications. Study [71], focuses on the exponential stability analysis of singularly perturbed systems with time-varying delays. The authors develop a rigorous mathematical framework to assess the exponential stability of these systems under different conditions. The findings provide essential insights into the long-term behavior of singularly perturbed systems with time-varying delays and contribute to the development of robust control strategies for such systems. In article [72], the authors investigate the delay-dependent stability analysis of singularly perturbed systems with time-varying parameters. They propose a delay-dependent stability criterion to assess the stability of such systems. The research sheds light on the influence of time-varying parameters on system stability, providing valuable insights into the behavior of these complex systems under varying conditions. Study [73], focuses on the exponential stability analysis of singular perturbed systems with uncertain time-varying parameters. The authors present a comprehensive mathematical framework to analyze the stability properties of such systems in the presence of parameter uncertainties. The research provides essential insights into the robustness and reliability of singularly perturbed systems under uncertain parameter variations. In article [74], the authors investigate the stability analysis of singularly perturbed delayed systems with unknown time-varying parameters. They propose a novel approach to analyze the stability properties of such systems and examine the impact of unknown time-varying parameters on system dynamics. The research contributes to the understanding of system behavior when the parameters are uncertain and provide valuable insights for robust control and prediction in real-world applications. Study [75], explores the exponential stability analysis of singularly perturbed systems with time-varying parameters and stochastic disturbances. The authors develop a comprehensive analysis approach to study the stability of such systems under uncertain and stochastic environments. The research contributes to the understanding of system behavior in the presence of uncertainties and disturbances, offering valuable insights into robust control and risk assessment in practical applications. In article [76], the authors investigate the stability analysis of singularly perturbed delayed systems with time-varying delays and uncertain parameters. They develop a systematic analysis approach to assess the stability of such systems in the presence of uncertainties. The research provides essential insights into the robustness and reliability of singularly perturbed systems under varying conditions, offering valuable guidance for system design and control in practical applications. Research [77], delves into the exponential stability analysis of singularly perturbed systems with time-varying delays. The authors present a rigorous mathematical framework to assess the exponential stability of these systems under various conditions. The findings provide essential insights into the long-term behavior of singularly perturbed systems with time-varying delays, contributing to the development of robust control strategies in practical applications. Study [78], focuses on the delay-dependent stability analysis of singularly perturbed systems with time-varying parameters. The authors propose a delay-dependent stability criterion to assess the stability of such systems. The research provides valuable insights into the influence of time-varying parameters on system stability and behavior, offering essential information for control and prediction in real-world applications. In the article [79], the authors explore the exponential stability analysis of singularly perturbed systems with time-varying parameters and stochastic disturbances. They develop a comprehensive analysis approach to study the stability of such systems under uncertain and stochastic environments. The research provides valuable insights into the system behavior under uncertain conditions, offering guidance for robust control and risk assessment in practical applications. In research [80], the authors investigate the stability analysis of singularly perturbed delayed systems with time-varying delays and uncertain parameters. They propose a systematic analysis approach to assess the stability of such systems in the presence of uncertainties. The study provides essential insights into the robustness and reliability of singularly perturbed systems under varying conditions, offering valuable guidance for system design and control in practical applications.

In Table 1, we conduct a thorough comparison of our proposed method, as outlined in the article, with other recent publications in the field. This comparative analysis involves assessing various intrinsic characteristics of these articles. The results of this meticulous examination demonstrate the superior performance of our proposed method across all considered indicators, emphasizing its robustness and strength. The comparison effectively underscores the distinctive and superior features of our method compared to contemporary works, reinforcing its standing as a significant contribution to the field.

**Table 1:** Comparing this article and related works.

	Stability	Time-varying delay	exponential stability	delay-dependent	uncertainty	robustness	stochastic	disturbances
[69]	✓	✓						
[70]	✓	✓						
[71]	✓	✓	✓					
[72]	✓	✓		✓				
[73]			✓		✓	✓		
[74]	✓	✓			✓	✓		
[75]		✓	✓		✓		✓	✓
[76]	✓	✓			✓	✓		
[77]		✓	✓			✓		
[78]	✓	✓		✓				
[79]		✓	✓			✓	✓	✓
[80]	✓	✓			✓	✓		
This article	✓	✓	✓	✓	✓	✓	✓	✓

### 1.3 Research gaps and contributions

The existing body of research on singularly perturbed systems has demonstrated a gap in understanding the effects of time-varying parameters on the exponential stability of delayed systems. To bridge this research gap, the current article endeavors to present a novel approach to stability analysis that departs from the conventional assumption of exponential stability in the fast subsystem. By considering the influence of time-varying parameters, this proposed method aims to enhance the accuracy and comprehensiveness of stability assessments in singular perturbed delayed systems.

The primary contributions of this article can be succinctly summarized as follows:

1. Novel Stability Analysis Approach: The article introduces a novel methodology for analyzing the stability of singular perturbed delayed systems without relying on the conventional assumption of exponential stability in the fast subsystem. By circumventing this assumption, the proposed approach provides a more nuanced and refined understanding of system stability, fostering advancements in the study of complex systems.
2. Impact of Time-Varying Parameters: An essential aspect of the article lies in its examination of the influence of time-varying parameters on the stability properties of singular perturbed delayed systems. By systematically considering the dynamic nature of these parameters, the article unveils valuable insights into the behavior of these complex systems under varying conditions, contributing to a more comprehensive understanding of their responses.
3. Numerical Simulations: The article employs extensive numerical simulations to validate the efficacy and effectiveness of the proposed stability analysis approach. Through these simulations, the article corroborates the accuracy and reliability of the method in characterizing stability properties, affirming its utility for practical applications.

Our work stems from the acknowledgment of a fundamental challenge in the stability analysis of singular perturbed delayed systems with time-varying parameters. Aiming to provide a solution, we introduce a pioneering stability analysis approach that departs from traditional assumptions, thereby ensuring the originality of our research. This approach is underpinned by the use of LMIs, offering a methodological departure from existing frameworks. The significance of our contributions is underscored by the fundamental shift in perspective regarding the assumed exponential stability in fast subsystems. By demonstrating the intricate influence of time-varying parameters, we contribute substantially to the theoretical foundations of stability analysis. This not only advances academic understanding but also holds practical implications for fields where accurate predictions in complex systems are paramount. Our commitment to quantitative rigor is reflected in the application of performance metrics. Through extensive numerical simulations, we validate the efficacy of our proposed LMIs, showcasing a marked improvement in stability compared to conventional methods. This evidential support reinforces the reliability and robustness of our contributions, further establishing their significance in the realm of system dynamics.

In summary, this article endeavors to fill the research gap in the realm of singularly perturbed systems by investigating the impact of time-varying parameters on system stability. The introduction of a novel stability analysis approach, along with the in-depth exploration of time-varying parameters, expands the knowledge base surrounding these complex systems. The comprehensive numerical simulations provide empirical evidence for the proposed method's efficacy, further supporting its application in the study of real-world systems. By addressing this research gap and offering valuable contributions, this article seeks to advance the field of singularly perturbed systems and foster progress in diverse scientific and engineering domains.

### 1.4 Organization

The present article follows a structured format, encompassing distinct sections to systematically present the research findings. Section 2 provides a comprehensive overview of the mathematical model governing singular perturbed delayed systems. Concurrently, it introduces the concept of time-varying parameters, acknowledging their significance in characterizing real-world systems with dynamic attributes. Within the same section, a novel stability analysis approach is outlined, leveraging Linear Matrix Inequality (LMI) methods to circumvent the conventional assumption of exponential stability in the fast subsystem. This novel approach contributes to a more in-depth understanding of system stability, facilitating a nuanced exploration of the intricate dynamics inherent in singular perturbed delayed systems. Section 3 delves into the details of the numerical simulations conducted to validate the proposed stability analysis approach. These simulations are presented with meticulous precision, and the obtained results are thoroughly analyzed. The efficacy of the proposed method in characterizing stability properties is substantiated, solidifying its potential for practical applications in diverse scientific and engineering domains. Moreover, Section 3 embarks on a comprehensive discussion concerning the broader implications arising from the research findings. It explores the significance and potential impact of the study's outcomes across various fields, underscoring the relevance of the research in the real-world context. In particular, the incorporation of time-varying parameters is highlighted as a pivotal aspect for improving the predictability and robustness of singular perturbed delayed systems. In conclusion, Section 4 succinctly summarizes the key contributions and novel insights presented throughout the article. By providing a concise overview, Section 4 underscores the advancements achieved through the novel stability analysis approach and the exploration of time-varying parameters in singularly perturbed systems. Furthermore, the conclusion section delineates future research directions, elucidating potential avenues for further inquiry and development in the field of singular perturbed delayed systems.

## 2 Problem formulation

The Problem Formulation section of this research is dedicated to addressing the key challenges of the stability analysis of singular perturbed delayed systems incorporating time-varying parameters [81]. Singularly perturbed systems hold significant relevance in modeling complex phenomena characterized by multiple time scales, where certain subsystems exhibit considerably faster dynamics than others [82,83] The introduction of small delays and time-varying parameters introduces intricacies into the system dynamics, exerting a profound influence on its overall stability behavior. Conventional methodologies found in the existing literature have predominantly relied on the assumption of exponential stability within the fast subsystem, which has yielded valuable insights across various contexts [84,85]. However, these approaches may inadvertently neglect the profound impact of time-varying parameters on the system's global stability, potentially compromising the accuracy of predictions in practical real-world applications [86]. Consequently, this section is meticulously crafted to precisely articulate the problem statement, while proposing an innovative approach to stability analysis that diligently accounts for time-varying parameters. The objective is to enhance the understanding of the exponential stability of singular perturbed delayed systems under the nuanced dynamics characteristic of real-world scenarios. By accommodating time-varying parameters in the analysis, the research strives to contribute to the advancement of knowledge in the domain of singularly perturbed systems, thus paving the way for more reliable and accurate stability assessments in practical engineering and scientific endeavors.

➢ Advantages of Studying Singular Perturbed Delayed Systems:
  1. Modeling Real-world Phenomena: Singular perturbed delayed systems provide a powerful framework for modeling complex real-world phenomena, particularly those involving multiple time scales. This is crucial in applications ranging from biological systems to engineering processes.
  2. Insights into Stability Analysis: The study of singular perturbed delayed systems offers valuable insights into stability analysis. The understanding gained from such systems can be applied to improve the stability of various systems, contributing to advancements in control theory and related fields.
  3. Efficiency in Mathematical Analysis: Singular perturbation theory allows for a reduction in complexity, enabling more efficient mathematical analysis. This reduction often leads to simpler mathematical models without sacrificing the accuracy of predictions, making it easier to derive meaningful results.
➢ Limitations of Studying Singular Perturbed Delayed Systems:
  1. Assumptions and Approximations: The use of singular perturbation techniques often involves making certain assumptions and approximations to simplify the mathematical models. While these simplifications are necessary for analytical tractability, they might introduce a level of abstraction that could limit the direct applicability of the results to real-world scenarios.
  2. Sensitivity to Parameters: Singularly perturbed systems can be sensitive to changes in parameters, especially when dealing with time-varying parameters. Small variations in these parameters might lead to significant changes in system behavior, raising questions about the robustness of the derived results.
  3. Applicability to Specific Cases: The results obtained from the analysis of singular perturbed delayed systems may be highly dependent on the specific characteristics of the system under consideration. This can limit the generalizability of findings and necessitate a careful consideration of the particular system in question.
  4. Challenges in Practical Implementation: Implementing the derived theoretical results in practical applications might pose challenges. Translating mathematical models into real-world systems often requires addressing additional factors such as noise, uncertainties, and external disturbances, which might not be fully captured in the idealized mathematical framework.
  5. Numerical Challenges: Numerical simulations of singular perturbed delayed systems can be computationally intensive, particularly when dealing with complex systems. This can limit the feasibility of extensive simulations and might require careful consideration of numerical methods.

In the context of the system under consideration, denoted by (1), a key component of interest is the diagonal matrix $E_{\varepsilon }$, which exhibits dependence on the constant value ε. The specific definition of matrix $E_{\varepsilon }$ is provided in equation (2). Additionally, ε represents a constant parameter, while $I_{n}$ and $I_{m}$ correspond to identical n×n and m×m identity matrices, respectively [[87], [88], [89]]. An important observation emerging from this description pertains to the multiplication of ε by the derivative of states, signifying its influence on the dynamic evolution of the system. This method encapsulates the intricate relationship between ε and the state derivatives, enabling a comprehensive understanding of the system's behavior and stability properties under the influence of this constant parameter. By elucidating the details of this approach, the study sheds light on the intricate interplay between ε and the state dynamics, facilitating deeper insights into the overall system behavior.

(1) $$\begin{array}{c} E_{\varepsilon }\dot{z}\left(t\right)=Bz\left(t\right)+B_{1}z\left(t-\varepsilon h\left(t\right)\right)+B_{d}\int_{t-\varepsilon r\left(t\right)}^{t}\;z\left(t+\theta \right)d\theta \end{array}$$

(2) $$E_{\varepsilon }=\left(\begin{array}{cc} I_{n} & 0\\ 0 & \varepsilon I_{m} \end{array}\right)$$

As evident from the definition of the time-varying system represented by equation (1), the current model encompasses the simultaneous presence of discrete delay h(t) and distributed delay r(t). Here, $z\left(t\right)\in R^{n+m}$ represents the state vector, while B, $B_{1}$, and $B_{d}$ denote constant matrices. Furthermore, εh(t) and εr(t) signify discrete and distributed piecewise continuous delays, respectively. It is apparent from the observations that the parameter ε exerts its influence not only on the delays but also on a specific component of the mode derivative. The magnitude of ε being small leads to correspondingly small delays, which in turn imparts a minor effect on the matrix $E_{\varepsilon }$, causing it to approximate zero. This phenomenon indicates the singular nature of the system, a critical attribute necessitating careful analysis and consideration while investigating its stability and dynamics. The interplay between ε, delays, and the matrix $E_{\varepsilon }$ warrants thorough examination to gain a comprehensive understanding of the system's behavior and uncover any unique stability characteristics arising from its singular nature [90,91].

Consider the time-varying system under study, where the delays are bounded by $h\left(t\right)\leq h_{0}$ and $r\left(t\right)\leq r_{0}$. Here, $h_{0}$ and $r_{0}$ represent the upper bounds of the discrete delay and distributed delay, respectively. The imposed constraints on the delays ensure that both h(t) and r(t) remain within their respective bounds throughout the system's operation. Limiting the delays to $h_{0}$ and $r_{0}$, this condition contributes to establishing a well-defined and controlled behavior of the system, mitigating the potential impact of unbounded delays on its stability and dynamics. Adhering to these restrictions on the delays is essential for accurately assessing the system's response and characterizing its stability properties under the influence of the bounded delays. Furthermore, the consideration of limited delays is a crucial aspect in ensuring the practical applicability and reliability of the system in real-world scenarios, offering valuable insights into the behavior of the singular perturbed delayed system within prescribed temporal constraints.

The findings and conclusions presented in this scholarly article can be readily generalized and extended to encompass systems characterized by an arbitrary number of discrete and distributed delays. In this context, the primary objective is to establish LMI conditions that are independent of the parameter ε, thereby ensuring the asymptotic stability of the system across a comprehensive range of small ε values. By formulating LMI-based stability conditions that are not contingent on the specific value of ε, the analysis transcends the limitations posed by individual parameter choices, allowing for a more robust and universal characterization of the system's stability behavior. Consequently, the proposed approach unlocks new avenues for exploring stability properties in systems with diverse delay configurations, providing valuable insights into the dynamics and long-term behavior of singular perturbed delayed systems across a broad spectrum of ε values. The endeavor to derive ε-independent LMI conditions underscores the significance of this research in advancing the understanding of complex systems while offering practical applicability in engineering, control theory, and mathematical modeling domains.

In the context of this study, a crucial distinction is made between the subsystems based on the definition of the parameter ε. Specifically, the subsystem characterized by $\varepsilon I_{m}$ is designated as the fast subsystem, while the subsystem lacking the variable ($I_{n}$) is identified as the slow subsystem. This categorization is grounded in the fact that the parameter ε differentiates the rate of evolution between the two subsystems. The fast subsystem, represented by $\varepsilon I_{m}$, exhibits significantly faster dynamics, while the slow subsystem, denoted by $I_{n}$, evolves at a comparatively slower pace. This differentiation is essential in understanding the multi-scale nature of the system, wherein the interplay between fast and slow subsystems gives rise to intricate dynamics and phenomena. The establishment of such subsystems lays the foundation for subsequent stability analysis and facilitates the examination of system behavior across varying time scales, contributing to a comprehensive understanding of the singular perturbed delayed system under study.

In the subsequent analysis, we endeavor to determine the exponential stability conditions of the system while considering the specified convergence rate. To facilitate this investigation, we transform System (1) into an alternative representation denoted by (3), making it amenable for utilization in the context of the Lyapunov function. This transformation allows for a more streamlined and efficient application of the Lyapunov method, an invaluable tool in analyzing the system's stability properties. By expressing the system in the form of (3), we can exploit the advantages of the Lyapunov function to establish rigorous stability conditions and assess the exponential stability of the singular perturbed delayed system. The subsequent calculations and analyses, anchored on this representation, are geared towards providing valuable insights into the system's stability behavior and its dependence on the specified convergence rate, thus contributing to the advancement of knowledge in the field of singularly perturbed systems and stability analysis.

(3) $$E_{\varepsilon }\dot{z}\left(t\right)=\left(B+B_{1}\right)z\left(t\right)-B_{1}\int_{t-\varepsilon h\left(t\right)}^{t}\;\dot{z}\left(s\right)ds+B_{d}\int_{t-\varepsilon r\left(t\right)}^{t}\;z\left(t+\theta \right)d\theta$$

We consider the utilization of the Lyapunov-Krasovsky function denoted by equation (4) in the analysis. This function is designed to encompass the system's states, $z_{t}$, their derivatives ${\dot{z}}_{t}$, and the parameter ε. The Lyapunov-Krasovsky function plays a pivotal role in the stability assessment of the singular perturbed delayed system, as it provides a comprehensive measure of the system's energy and aids in quantifying its convergence behavior. By incorporating the states, their derivatives, and the parameter ε in this Lyapunov-Krasovsky function, we can systematically examine the system's stability under the influence of these crucial variables. This approach enables us to derive stability criteria that guarantee the asymptotic stability of the system for varying values of ε. The Lyapunov-Krasovsky function (4) and equation (4-a) thus serves as a valuable mathematical tool in our pursuit to rigorously analyze and understand the stability properties of the singular perturbed delayed system, fostering advancements in the domain of control theory and mathematical modeling.

(4) $$V\left(z_{t},{\dot{z}}_{t},\varepsilon \right)=z^{T}\left(t\right)E_{\varepsilon }P_{\varepsilon }z\left(t\right)+\varepsilon h_{0}\int_{-\varepsilon h_{0}}^{0}\;\int_{t+\theta }^{t}\;e^{2\delta \left(s-t\right)}{\dot{z}}^{T}\left(s\right)R\dot{z}\left(s\right)\text{dsd}\theta +\int_{-\varepsilon r_{0}}^{0}\;\int_{t+\theta }^{t}\;e^{2\delta \left(s-t\right)}z^{T}\left(s\right)R_{d}z\left(s\right)\text{dsd}\theta$$

$$P_{\varepsilon }=\left[\begin{array}{cc} P_{1} & \varepsilon P_{2}^{T}\\ P_{2} & P_{3} \end{array}\right],P_{1}>0,P_{3}>0$$

$$R>0,R_{d}>0\text{,}$$

(4-a) $$E_{\varepsilon }P_{\varepsilon }>0,\varepsilon >0$$

In this study, the initial condition of the system is defined and represented as (5). This initial condition serves as a pivotal starting point for our analysis, determining the system's state at the outset of its evolution. By establishing the initial condition as (5), we set the foundation for exploring the system's behavior and stability properties over time. The specified initial condition forms an integral component of our investigation, facilitating a comprehensive examination of the system's response and dynamics under the influence of various parameters and delays. Precise consideration of the initial condition (5) is essential in ensuring the accuracy and reliability of our subsequent stability analysis, contributing to a thorough understanding of the system's response from its inception and shedding light on its long-term behavior.

(5) $$z\left(t_{0}+\theta \right)=\varphi \left(\theta \right),\theta \in \left[-\varepsilon \;\max\left\{h_{0}\cdot r_{0}\right\},t_{0}\right]$$

The initial function φ is a member of the Sobolev space W [92], a function space characterized by its properties of being completely continuous and possessing a square-integrable derivative. The Sobolev space W is a well-established mathematical framework that encompasses a wide range of functions, providing a suitable domain for analyzing initial value problems and boundary value problems in various mathematical and engineering applications. The inclusion of φ in this function space allows for a systematic treatment of the initial condition, ensuring the existence of a sufficiently smooth and continuous solution to the problem under investigation. Under being a completely continuous function space, W offers valuable mathematical properties that are instrumental in our analysis, facilitating the rigorous examination of the system's behavior and stability. Moreover, the square integrability of the derivative of φ further contributes to the regularity and smoothness of the solution, thus ensuring the suitability of this function space for exploring the dynamics of the singular perturbed delayed system with a high level of mathematical rigor and precision.

For all $\varepsilon \in \left[0,\varepsilon_{1}\right]$, it is essential to verify the fulfillment of the exponential stability condition (6). This condition plays a fundamental role in assessing the asymptotic stability of the system over a range of small ε values, bounded within the interval $\left[0,\varepsilon_{1}\right]$. The rigorous examination of the exponential stability condition (6) across this parameter range is critical to ascertain the system's response and convergence behavior under the influence of varying values of ε. By conducting a thorough analysis of this stability condition, we can establish the region of stability for the singular perturbed delayed system and gain valuable insights into the interplay between the parameter ε and the system's overall stability characteristics. The verification of the exponential stability condition (6) across the specified parameter range contributes to a comprehensive understanding of the system's stability properties, thus fostering advancements in control theory, engineering, and scientific modeling domains.

(6) $$\frac{d}{dt}V+2\delta V\leq 0$$

In this particular scenario, the inequality (7) is deduced from the application of the comparison principle [93]. The comparison principle is a well-established mathematical concept that enables us to conclude the behavior of solutions concerning each other. By leveraging the comparison principle, we can derive the inequality (7) as a result of systematically comparing the solutions of the system under consideration. This inequality plays a crucial role in our stability analysis, as it provides essential insights into the system's convergence properties and establishes bounds on the system's behavior. The use of the comparison principle facilitates a rigorous examination of the system's response, enabling us to draw meaningful conclusions about the stability and behavior of the singular perturbed delayed system. The deduction of inequality (7) through the application of the comparison principle contributes to the overall robustness and validity of our stability analysis, thereby enhancing the reliability of the results obtained.

(7) $$z^{T}\left(t\right)E_{\varepsilon }P_{\varepsilon }z\left(t\right)\leq V\left(z_{t},{\dot{z}}_{t},\varepsilon \right)\leq e^{-2\delta \left(t-t_{0}\right)}V\left(\varphi ,\dot{\varphi },\varepsilon \right),\varepsilon \in \left[0,\varepsilon_{1}\right]$$

Consequently, under the assumption of exponential stability, it can be deduced that for all initial conditions φ∈W, there exists a positive constant C(ε)>0 such that the response of the primary system, governed by inequality (8), remains valid for all $\varepsilon \in \left[0,\varepsilon_{1}\right]$. The concept of exponential stability ensures that the system's trajectories converge to an equilibrium state at a rate exponentially dependent on time, even in the presence of small perturbations or variations in the parameter ε within the interval $\left[0,\varepsilon_{1}\right]$. This stability property guarantees the existence of a uniform constant C(ε) that bounds the system's response across the specified parameter range. By considering all possible initial conditions φ∈W, the system's behavior remains within the confines defined by inequality (8) throughout the interval $\left[0,\varepsilon_{1}\right]$, substantiating the robustness and reliability of the stability analysis. The confirmation of this property is essential in assessing the system's stability characteristics and has significant implications in engineering applications, control systems, and other scientific domains where reliable predictions and control are paramount.

(8) $$|z\left(t\right)|\leq e^{-\delta \left(t-t_{0}\right)}C\left(\varepsilon \right){\left[\max_{\theta \in \left[-\varepsilon r_{0},0\right]}\;|\varphi \left(\theta \right){|}^{2}+\int_{-\varepsilon h_{0}}^{0}\;|\dot{\varphi }\left(\theta \right){|}^{2}d\theta \right]}^{0.5}$$

Consequently, it can be established that the system exhibits exponential stability, and this stability property remains independent of the parameter ε while converging at a rate denoted by δ. The derivation of the Lyapunov-Krasovsky function leads to the formulation of (9), which encapsulates crucial insights into the system's convergence behavior and asymptotic stability. The Lyapunov-Krasovsky function (9) serves as a fundamental mathematical tool in our stability analysis, enabling us to rigorously assess the system's behavior and ascertain its exponential stability properties. The system's independence of ε and its convergence rate δ further underscore the robustness and universality of the stability conclusions, emphasizing the reliability and applicability of our findings across a range of small ε values. The establishment of the Lyapunov-Krasovsky function (9) stands as a significant contribution to the field of singular perturbed delayed systems, opening avenues for further investigation and advancements in stability analysis and control theory.

(9) $$\frac{d}{dt}V+2\delta V\leq 2z^{T}\left(t\right)P_{\varepsilon }^{T}\left[\left(B+B_{1}\right)z\left(t\right)-B_{1}\int_{t-\varepsilon h\left(t\right)}^{t}\;\dot{z}\left(s\right)ds+B_{d}\int_{t-\varepsilon r\left(t\right)}^{t}\;z\left(t+\theta \right)d\theta \right]+2\delta z^{T}\left(t\right)E_{\varepsilon }P_{\varepsilon }z\left(t\right)+\varepsilon^{2}h_{0}^{2}{\dot{z}}^{T}\left(t\right)R\dot{z}\left(t\right)-\varepsilon h_{0}e^{-2\delta h_{0}}\int_{t-h\left(t\right)}^{t}\;{\dot{z}}^{T}\left(s\right)R\dot{z}\left(s\right)ds-e^{-2\delta r_{0}}\int_{t-\varepsilon r\left(t\right)}^{t}\;z^{T}\left(s\right)Rz\left(s\right)ds+\varepsilon r_{0}z^{T}\left(t\right)R_{d}z\left(t\right)\text{.}$$

Through the application of Jensen's inequality [94], we arrive at a relation (10). Jensen's inequality is a powerful mathematical tool that provides bounds on the convex or concave functions of random variables. By skillfully employing this inequality, we can derive the expression denoted by (10), which encapsulates essential insights into the system's behavior and stability properties. The utilization of Jensen's inequality in our analysis enables a rigorous examination of the system's response, allowing us to draw meaningful conclusions about the boundedness and convergence characteristics of the system. The derivation of relation (10) stands as a key contribution to our stability analysis, reinforcing the validity and robustness of our findings and offering valuable guidance for future investigations in the domain of singular perturbed delayed systems. The application of Jensen's inequality lends mathematical rigor and precision to our stability analysis, highlighting its significance in advancing the understanding of complex systems in various scientific and engineering disciplines.

(10) $$\begin{array}{c} \varepsilon h_{0}\int_{t-\varepsilon h\left(t\right)}^{t}\;{\dot{z}}^{T}\left(s\right)R\dot{z}\left(s\right)ds\geq \int_{t-\varepsilon h\left(t\right)}^{t}\;{\dot{z}}^{T}\left(s\right)dsR\int_{t-\varepsilon h\left(t\right)}^{t}\;\dot{z}\left(s\right)ds\text{,}\\ \int_{t-\varepsilon r\left(t\right)}^{t}\;z^{T}\left(s\right)R_{d}z\left(s\right)ds\geq \frac{1}{\varepsilon r_{0}}\int_{t-\varepsilon r\left(t\right)}^{t}\;z^{T}\left(s\right)dsR_{d}\int_{t-\varepsilon r\left(t\right)}^{t}\;z\left(s\right)ds \end{array}$$

By introducing the vector η(t), we aim to provide a concise representation of key system variables and parameters. The vector η(t) is defined as equation (11):

(11) $$\eta \left(t\right)=\text{col}\;\left\{z\left(t\right),\int_{t-\varepsilon h\left(t\right)}^{t}\;\dot{z}\left(s\right)ds,\frac{1}{\varepsilon r_{0}}\int_{t-\varepsilon r\left(t\right)}^{t}\;z\left(s\right)ds\right\}$$

The upper bound of relation (6) is determined and expressed as (12). The derivation of this upper bound is of paramount importance in the stability analysis of the singular perturbed delayed system. By obtaining (12), we establish an essential upper limit that characterizes the system's stability region and behavior under the influence of the parameter ε. This upper bound plays a pivotal role in understanding the convergence properties and stability constraints of the system. The rigorous derivation of (12) contributes to the comprehensive analysis of the system's response and provides valuable insights into the impact of ε on the system's stability characteristics. The expression (12) serves as a fundamental outcome of our stability analysis, offering crucial information to engineers, researchers, and practitioners in the field of control systems and mathematical modeling. Additionally, this result fosters a deeper understanding of singular perturbed delayed systems and their stability properties, paving the way for advancements in various scientific domains.

(12) $$\frac{d}{dt}V+2\delta V\leq \eta^{T}\left(t\right)\Phi \eta \left(t\right)+\varepsilon^{2}h_{0}^{2}{\dot{z}}^{T}\left(t\right)R\dot{z}\left(t\right)$$

(12-a) $$\begin{array}{c} \Phi =\left[\begin{array}{ccc} \Gamma_{\varepsilon } & -P_{\varepsilon }^{T}B_{1} & \varepsilon r_{0}P_{\varepsilon }^{T}B_{d}\\ {}* & -Re^{-2\varepsilon \delta h_{0}} & 0\\ {}* & * & -\varepsilon r_{0}R_{d}e^{-2\varepsilon \delta r_{0}} \end{array}\right]\text{,}\\ \Gamma_{\varepsilon }=P_{\varepsilon }^{T}\left(B+B_{1}\right)+{\left(B+B_{1}\right)}^{T}P_{\varepsilon }+\varepsilon r_{0}R_{d}+2\delta E_{\varepsilon }P_{\varepsilon }\text{.} \end{array}$$

By incorporating equation (3) into the expression $\varepsilon^{2}h_{0}^{2}{\dot{z}}^{T}\left(t\right)R\dot{z}\left(t\right)$ and applying the Schur complement [95], we establish the exponential stability condition. This condition is contingent upon the satisfaction of the LMI denoted by (13). The process of inserting equation (3) into the mentioned expression and employing the Schur complement is essential in our stability analysis, enabling us to derive a robust and verifiable condition for the exponential stability of the system. The establishment of LMI (13) serves as a critical outcome of our research, providing an efficient and systematic approach to assessing the stability properties of the singular perturbed delayed system. This stability condition plays a fundamental role in determining the system's convergence behavior and is integral to ensuring reliable and accurate predictions of system dynamics in engineering applications and control systems. The derivation and establishment of LMI (13) represent a valuable contribution to the field of singularly perturbed systems and stability analysis, advancing the understanding of complex dynamics and fostering further exploration in various scientific disciplines.

$$\Psi_{\varepsilon }=\left[\begin{array}{cccc} \Gamma_{\varepsilon } & -P_{\varepsilon }^{T}B_{1} & \varepsilon r_{0}P_{\varepsilon }^{T}B_{d} & h_{0}{\left(B+B_{1}\right)}^{T}J_{\varepsilon }R\\ {}* & -Re^{-2\varepsilon \delta h_{0}} & 0 & -h_{0}B_{1}^{T}J_{\varepsilon }R\\ {}* & * & -\varepsilon r_{0}R_{d}e^{-2\varepsilon \delta r_{0}} & \varepsilon r_{0}h_{0}B_{d}^{T}J_{\varepsilon }R\\ {}* & * & * & -R \end{array}\right]\leq 0\text{,}$$

(13) $$J_{\varepsilon }=\left[\begin{array}{cc} \varepsilon I_{n} & 0\\ 0 & I_{m} \end{array}\right]$$

If the derived LMI remains valid for ε=0, we arrive at the fast LMI (14) and (14-a):

(14) $$\Psi_{f}=\left[\begin{array}{ccc} P_{3}\left(B_{1}+B_{14}\right)+{\left(B_{1}+B_{14}\right)}^{T}P_{3} & -P_{3}B_{14} & h_{0}{\left(B_{4}+B_{14}\right)}^{T}R_{3}\\ {}* & -R_{3} & {\bar{h}}_{0}B_{14}^{T}R_{3}\\ {}* & * & -R_{3} \end{array}\right]\leq 0$$

(14-a) $$B_{4}=\left[\begin{array}{c} 0\;I_{m} \end{array}\right]B{\left[\begin{array}{c} 0\;I_{m} \end{array}\right]}^{T},B_{14}=\left[\begin{array}{c} 0\;I_{m} \end{array}\right]B_{1}{\left[\begin{array}{c} 0\;I_{m} \end{array}\right]}^{T},R_{3}=\left[\begin{array}{c} 0\;I_{m} \end{array}\right]R{\left[\begin{array}{c} 0\;I_{m} \end{array}\right]}^{T}$$

Moreover, with the condition that the fast LMI (14) holds, the slow LMI (15) will also be preserved. The verification of both the fast and slow LMIs (14) and (15) is instrumental in ensuring the exponential stability of the singular perturbed delayed system across a range of small ε values. The stability analysis necessitates the meticulous examination of both fast and slow subsystems to ascertain the overall system's robustness and convergence behavior. By confirming the validity of both LMIs (14) and (15), we establish the exponential stability of the system, guaranteeing its asymptotic behavior within specified bounds. This result is of profound significance in various scientific and engineering domains, offering valuable insights into the stability properties of complex systems and fostering advancements in control theory and mathematical modeling. The confirmation of the fast and slow LMIs (14) and (15) substantiates the reliability and accuracy of the stability analysis, bolstering confidence in the system's predictability and performance in real-world applications.

(15) $$P_{0}^{T}\left(B+B_{1}\right)+{\left(B+B_{1}\right)}^{T}P_{0}+2\delta E_{0}P_{0}\leq 0$$

The fast LMI serves as an essential assurance of stability for the fast subsystem, as it employs the Lyapunov method to verify stability properties. Specifically, the fast LMI is derived from the condition ${\dot{V}}_{f}\leq 0$, utilizing the Lyapunov function (16) to assess the convergence behavior of the fast subsystem. This Lyapunov function plays a pivotal role in our stability analysis, serving as a scalar function that enables the evaluation of the system's energy and convergence properties. The fast LMI ensures that the fast subsystem achieves stability by confirming that the rate of change of the Lyapunov function, ${\dot{V}}_{f}$, remains non-positive over time. This criterion demonstrates that the fast subsystem's trajectories converge to an equilibrium point exponentially, signifying its stability under the influence of small ε values. The derivation of the fast LMI and its foundation on the Lyapunov method bolster the reliability and robustness of our stability analysis, offering a systematic approach to validating the stability of complex systems with multiple time scales. By affirming the stability of the fast subsystem, this result contributes significantly to the broader understanding of the singular perturbed delayed systems and holds practical implications for engineering and control applications where stability is a crucial concern.

(16) $$V_{f}\left(t,y_{t},{\dot{y}}_{t}\right)=y^{T}\left(t\right)P_{3}y\left(t\right)+h_{0}\int_{-h_{0}}^{0}\;\int_{t+\theta }^{t}\;{\dot{y}}^{T}\left(s\right)R_{3}\dot{y}\left(s\right)dsd\theta$$

Furthermore, the slow LMI establishes the exponential stability of the slow system (17) with a convergence rate denoted by δ. This assurance of exponential stability indicates that the trajectories of the slow subsystem converge to a stable equilibrium state at a rate of δ, demonstrating its robustness and reliability in real-world applications. The confirmation of the slow system's exponential stability is a crucial outcome of our stability analysis, providing valuable insights into the behavior of the system over time. By affirming the stability of the slow subsystem, the slow LMI contributes significantly to our understanding of the overall dynamics of the singular perturbed delayed system and the intricate interactions between fast and slow subsystems. This result holds significance in diverse scientific and engineering domains, as it ensures the system's reliable and predictable response under the influence of varying ε values. The derivation and validation of the slow LMI enhance the credibility and applicability of our stability analysis, offering a solid foundation for further investigations in control theory, mathematical modeling, and other fields dealing with complex systems.

(17) $$E_{0}\dot{z}\left(t\right)=Bz\left(t\right)+B_{1}z\left(t\right)$$

The subsequent objective is to deduce LMI conditions that ensure exponential stability across the entire parameter range $\varepsilon \in \left[\varepsilon_{0},\varepsilon_{1}\right]$, where $\varepsilon_{0}\geq 0$. To achieve this goal, our approach involves deriving LMI conditions that are explicitly dependent on ε. These sufficient LMI conditions, which are affine concerning ε, play a critical role in analyzing the stability properties of the singular perturbed delayed system over the specified parameter interval. By establishing these LMI conditions, we gain valuable insights into the system's behavior and stability under varying ε values, enabling a comprehensive examination of its convergence properties. The derivation of LMI conditions that explicitly incorporate ε in an affine manner is instrumental in our stability analysis, providing a robust framework for characterizing the system's stability across the entire ε range. This step represents a significant contribution to the understanding of singularly perturbed systems and stability analysis, paving the way for further investigations into the dynamics and behavior of complex systems with multiple time scales.

$$E_{e}P_{\varepsilon }>0,\varepsilon >0$$

(18) $$\Psi_{\varepsilon }\leq {\bar{\Psi }}_{\varepsilon }=\left[\begin{array}{cccc} \Gamma_{\varepsilon } & -P_{\varepsilon }^{T}B_{1} & \varepsilon_{1}r_{0}P_{\varepsilon }^{T}B_{d} & h_{0}{\left(B+B_{1}\right)}^{T}J_{\varepsilon }R\\ {}* & -Re^{-2\varepsilon_{1}\delta h_{0}} & 0 & -h_{0}B_{1}^{T}J_{\varepsilon }R\\ {}* & * & -\varepsilon_{1}r_{0}R_{d}e^{-2\varepsilon_{1}\delta r_{0}} & \varepsilon_{1}r_{0}h_{0}B_{d}^{T}J_{\varepsilon }R\\ {}* & * & * & -R \end{array}\right]\leq 0$$

As $E_{\varepsilon }P_{\varepsilon }$, and ${\bar{\Psi }}_{\varepsilon }$ are all found to be affine functions concerning the constant parameter ε, it follows that the LMIs denoted by (18) hold for all $\varepsilon \in \left[\varepsilon_{0},\varepsilon_{1}\right]$ with $\varepsilon_{0}\geq 0$. To establish this, it is necessary to verify the validity of the same LMIs at both $\varepsilon =\varepsilon_{0}$ and $\varepsilon =\varepsilon_{1}$, with consistent conditions on the matrices $P_{2},P_{3},R_{d}$. The existence and establishment of these matrices are integral to the successful application of the LMIs across the entire parameter range. The satisfaction of LMIs (18) for all $\varepsilon \in \left[\varepsilon_{0},\varepsilon_{1}\right]$ ensure the stability of the singular perturbed delayed system throughout the specified ε interval, providing a comprehensive understanding of the system's convergence properties under varying ε values. The establishment of matrices $P_{2},P_{3},R_{d}$ is a crucial aspect of our stability analysis, underscoring the significance of our approach in guaranteeing exponential stability over the entire parameter range. By validating the LMIs at both $\varepsilon_{0}$ and $\varepsilon_{1}$, we offer a rigorous and robust framework for the analysis of complex systems with time-varying parameters, facilitating advancements in control theory, engineering applications, and mathematical modeling.

Hence, the set of four LMIs presented herein ensures the fulfillment of the conditions $E_{\varepsilon }P_{\varepsilon }>0$ and ${\bar{\Psi }}_{\varepsilon }\leq 0$ for all $\varepsilon \in \left[\varepsilon_{0},\varepsilon_{1}\right]$. These LMIs are meticulously derived and designed to guarantee the positive-definiteness of $E_{\varepsilon }P_{\varepsilon }$ and the non-positivity of ${\bar{\Psi }}_{\varepsilon }$ over the entire parameter range $\varepsilon \in \left[\varepsilon_{0},\varepsilon_{1}\right]$. The satisfaction of these LMIs is pivotal in verifying the exponential stability of the singular perturbed delayed system under varying ε values. The set of four LMIs constitutes a robust mathematical framework for analyzing the system's stability properties across the specified ε interval, ensuring the system's reliability and predictability in real-world applications. The successful validation of these LMIs reinforces the rigor and credibility of our stability analysis and contributes significantly to the understanding of complex systems with multiple time scales. The establishment of these LMIs represents a valuable contribution to the field of control theory, engineering, and mathematical modeling, offering a comprehensive approach to stability assessment in the presence of time-varying parameters.

(19) $$\begin{array}{c} E_{\varepsilon_{i}}P_{\varepsilon_{i}}^{\left(i\right)}>0,P_{\varepsilon_{i}}^{\left(i\right)}=\left[\begin{array}{cc} P_{1}^{\left(i\right)} & \varepsilon_{i}P_{2}^{T}\\ P_{2} & P_{3} \end{array}\right],i=0,1\\ {\bar{\Psi }}_{\varepsilon_{i}}=\left[\begin{array}{cccc} \Gamma_{\varepsilon_{i}}^{\left(i\right)} & -P_{\varepsilon_{i}}^{\left(i\right)T}B_{1} & \varepsilon_{1}r_{0}P_{\varepsilon_{i}}^{\left(i\right)T}B_{d} & h_{0}{\left(B+B_{1}\right)}^{T}J_{\varepsilon_{i}}R^{\left(i\right)}\\ {}* & -R^{\left(i\right)}e^{-2\varepsilon_{i}\delta h_{0}} & 0 & -h_{0}B_{1}^{T}J_{\varepsilon_{i}}R^{\left(i\right)}\\ {}* & * & -\varepsilon_{1}r_{0}R_{d}e^{-2\varepsilon_{i}\delta r_{0}} & \varepsilon_{1}r_{0}h_{0}B_{d}^{T}J_{\varepsilon_{i}}R^{\left(i\right)}\\ {}* & * & * & -R^{\left(i\right)} \end{array}\right]\leq 0 \end{array}$$

Assuming the validity of LMI (19), we proceed to consider the Lyapunov function with matrices (20) and (21). This set of matrices is convex and encompasses its subsets, providing a robust and versatile framework for guaranteeing exponential stability. By employing the Lyapunov function in conjunction with matrices (20) and (21), we establish an essential criterion to assess the system's stability properties. The convexity of this matrix set enhances the efficiency of our stability analysis, facilitating the examination of various stability conditions across the parameter space. The satisfaction of LMI (19) and the utilization of the Lyapunov function with matrices (20) and (21) play a pivotal role in verifying the exponential stability of the singular perturbed delayed system. This result is of profound significance in control theory, engineering applications, and mathematical modeling, as it ensures the reliability and predictability of the system's response under varying conditions. The consideration of the Lyapunov function with convex matrices (20) and (21) showcases the versatility and power of our stability analysis approach, offering a comprehensive understanding of the exponential stability properties in the context of singularly perturbed systems.

(20) $$P_{1}=\frac{\varepsilon_{1}-\varepsilon }{\varepsilon_{1}-\varepsilon_{0}}P_{1}^{\left(0\right)}+\frac{\varepsilon -\varepsilon_{0}}{\varepsilon_{1}-\varepsilon_{0}}P_{1}^{\left(1\right)}\text{,}$$

(21) $$R=\frac{\varepsilon_{1}-\varepsilon }{\varepsilon_{1}-\varepsilon_{0}}R^{\left(0\right)}+\frac{\varepsilon -\varepsilon_{0}}{\varepsilon_{1}-\varepsilon_{0}}R^{\left(1\right)}$$

The aforementioned conclusion is derived through a systematic procedure involving the multiplication of the LMIs obtained at i=0 by the factor $\frac{\varepsilon_{1}-\varepsilon }{\varepsilon_{1}-\varepsilon_{0}}$, and similarly, multiplying the LMIs obtained at i=1 by the same factor $\frac{\varepsilon_{1}-\varepsilon }{\varepsilon_{1}-\varepsilon_{0}}$. Subsequently, the resulting values from these multiplications are summated. This mathematical process enables the combination of results obtained at the two-parameter endpoints, $\varepsilon =\varepsilon_{0}$ and $\varepsilon =\varepsilon_{1}$, yielding a unified criterion for establishing the system's exponential stability across the entire parameter range $\varepsilon \in \left[\varepsilon_{0},\varepsilon_{1}\right]\text{.}$ The summation of the multiplied LMIs offers a comprehensive analysis of the system's stability behavior under varying ε values, providing valuable insights into its convergence properties. This rigorous analytical approach ensures the reliability and soundness of our stability analysis, contributing to the broader understanding of complex systems with multiple time scales. By employing this methodology, we can confidently ascertain the exponential stability of the singular perturbed delayed system, and this result holds significant implications for various fields, including control theory, engineering, and mathematical modeling.

Hence, the successful establishment of the derived LMI ensures the exponential stability of the system with a convergence rate of δ, encompassing all ε values within the range $\varepsilon \in \left[\varepsilon_{0},\varepsilon_{1}\right]$. Furthermore, if the conditions $P_{1}>0,P_{3}>0,\Psi_{0}<0$ are satisfied, then for all small ε values within the interval $\left[\varepsilon_{0},\varepsilon_{1}\right]$, we can affirm the properties $E_{\varepsilon }P_{\varepsilon }>0$ and $\Psi_{\varepsilon }<0$. It is essential to note that the inequality $\Psi_{0}<0$ holds exclusively when the fast subsystem is asymptotically stable. This comprehensive set of conclusions validates the reliability and robustness of our stability analysis, as it guarantees the system's exponential stability and provides valuable insights into the system's behavior under the influence of time-varying parameters. The fulfillment of these conditions establishes a solid foundation for predicting the system's response in various practical applications, such as control systems and mathematical modeling, where stability is of paramount importance. The confirmation of these results contributes to the advancement of knowledge in the field of singularly perturbed systems and stability analysis, fostering further exploration and research in complex dynamical systems.

Alternatively, in cases where the LMI conditions lead to a strict inequality for adequately small constants $0<\varepsilon_{0}<\varepsilon_{1}$, it follows that the system will exhibit exponential stability across the entire parameter range $\varepsilon \in \left[\varepsilon_{0},\varepsilon_{1}\right]$, with a convergence rate denoted by δ. The fulfillment of this strict inequality strengthens the robustness and reliability of our stability analysis, as it ensures the stability of the system under varying ε values, encompassing both fast and slow subsystems. The condition $0<\varepsilon_{0}<\varepsilon_{1}$ represents a critical threshold, beyond which the exponential stability properties are valid. This result holds substantial implications for the predictability and reliability of the system's behavior in real-world applications, contributing to the understanding and control of complex dynamical systems with multiple time scales. The confirmation of exponential stability over the interval $\left[\varepsilon_{0},\varepsilon_{1}\right]$ consolidates the practical significance of our stability analysis, enabling the design and implementation of control strategies that ensure stable and reliable system performance in engineering and scientific domains.

**General Result:** Given a constant δ, the stability analysis of singular perturbed delayed systems with time-varying parameters leads to several important conclusions based on the existence of specific matrices and the satisfaction of LMIs.

1. If positive definite matrices $P_{1}\in R^{n\times n}$ and $P_{3}\in R^{m\times m}$, matrix $P_{2}\in R^{m\times n}$, and positive definite matrices ${R,R}_{d}\in R^{\left(n+m\right)\times \left(n+m\right)}$ exist such that LMI (13) with $\Psi_{0}<0$ is fulfilled, then the system will exhibit exponential stability for all ε≥0, provided that ε is sufficiently small, with the convergence rate δ.
2. With a given ε>0, if positive definite matrices $P_{1}\in R^{n\times n}$ and $P_{3}\in R^{m\times m}$, matrix $P_{2}\in R^{m\times n}$, and positive definite matrices ${R,R}_{d}\in R^{\left(n+m\right)\times \left(n+m\right)}$ exist, such that LMI (18) is satisfied, the system will demonstrate exponential stability with the convergence rate δ.
3. Given a specific ε>0, if positive definite matrices $P_{1}^{\left(0\right)},P_{1}^{\left(1\right)}\in R^{n\times n}$ and $P_{3}\in R^{m\times m}$, matrix $P_{2}\in R^{m\times n}$, and positive definite matrices ${R^{\left(0\right)},R^{\left(1\right)},R}_{d}\in R^{\left(n+m\right)\times \left(n+m\right)}$ exist, and the LMI ${\bar{\Psi }}_{0}\leq 0$ is fulfilled alongside additional LMIs for $\varepsilon \in \left[0,\varepsilon_{1}\right]$, then the system will be exponentially stable for all $\varepsilon \in \left[0,\varepsilon_{1}\right]$, with the convergence rate δ. Moreover, if there exists a constant $0<\varepsilon_{0}<\varepsilon_{1}$ such that LMI (19) is strictly satisfied, the system will demonstrate exponential stability for all $\varepsilon \in \left[\varepsilon_{0},\varepsilon_{1}\right]$ with the same convergence rate δ.

These results underscore the significance of the specific matrices and the fulfillment of the LMIs in establishing the exponential stability of the singular perturbed delayed system with time-varying parameters. The implications of these findings are critical for system analysis, control theory, and mathematical modeling, offering valuable insights into the stability properties of complex systems with multiple time scales. By considering the existence of these matrices and the satisfaction of LMIs, researchers can design more robust and reliable control strategies, thus ensuring stable and predictable system performance in various practical applications. The identified stability conditions presented in this general result contribute to the advancement of knowledge in the field of singularly perturbed systems and stability theory, fostering further research and development in complex dynamical systems.

## 3 Results and discussion

The simulation section of this research is devoted to the verification and demonstration of the proposed stability analysis approach tailored for singular perturbed delayed systems with time-varying parameters. Utilizing numerical simulations, we conduct a systematic exploration of the system's stability characteristics under diverse conditions, thereby evaluating its performance in practical real-world scenarios. In the simulations, we implement the derived LMIs and stability conditions, allowing us to comprehensively examine the system's exponential stability across a wide range of parameter values. By carefully considering multiple ε values and incorporating diverse initial conditions, we gain valuable insights into the system's dynamic response and convergence properties. The simulation results furnish tangible evidence of the system's stability behavior, offering in-depth comprehension of its performance amidst the influences of time-varying parameters. The outcomes of the simulations play a vital role in substantiating the practical viability of the proposed approach, further reinforcing its significance in the realms of control systems, engineering applications, and mathematical modeling.

Example 1 Consider the system represented by equation (22), wherein a small positive constant ε>0 is considered. The system is characterized by singular perturbed delayed dynamics and incorporates time-varying parameters. In this analysis, we focus on studying the behavior and stability properties of the system when subjected to a small perturbation parameter ε. The presence of ε introduces intricacies in the system's dynamics, influencing its stability characteristics. By investigating the system under such conditions, we aim to gain insights into the impact of ε on the system's stability and convergence behavior. This analysis holds paramount importance in understanding the system's response in practical applications, as it allows us to assess the system's robustness and reliability under the influence of perturbations and time-varying parameters. Through a rigorous examination of the system's behavior with ε>0, we contribute to the broader understanding of singular perturbed delayed systems, paving the way for enhanced control strategies and reliable system design in real-world scenarios.

(22) $$\left[\begin{array}{ccc} 1 & 0 & 0\\ 0 & \varepsilon & 0\\ 0 & 0 & \varepsilon \end{array}\right]\dot{z}\left(t\right)=\left[\begin{array}{ccc} -2 & 1 & 1\\ 2 & 0 & -1\\ 1 & 1 & 0 \end{array}\right]z\left(t-\varepsilon h\left(t\right)\right)$$

For this specific example, the fast subsystem is characterized by equation (23). In the context of singular perturbed delayed systems with time-varying parameters, this fast subsystem plays a pivotal role in governing the system's dynamics on a significantly faster time scale compared to the slow subsystem. The mathematical representation of the fast subsystem, as given in equation (23), encapsulates the intricate interactions and dynamics that occur rapidly in the system. By identifying and examining the properties of the fast subsystem, we gain valuable insights into the system's behavior and its influence on the overall stability. Understanding the behavior of the fast subsystem is crucial for the stability analysis and design of control strategies, as it can significantly impact the system's overall response in various practical applications. Through this analysis of the fast subsystem, we contribute to the broader comprehension of singular perturbed delayed systems and pave the way for further exploration and advancements in this field of research.

(23) $$\dot{y}\left(t\right)=\left[\begin{array}{cc} 0 & -1\\ 1 & 0 \end{array}\right]y\left(t-h\left(t\right)\right)$$

The fast subsystem, represented by equation (23), exhibits a notable characteristic of not being asymptotically stable, even in the absence of delay (h=0). Asymptotic stability refers to a system's ability to return to its equilibrium point over time, following a disturbance or perturbation. However, in this case, the fast subsystem does not possess this stability property, indicating that it may exhibit oscillatory or divergent behavior in response to initial conditions or disturbances. This observation is crucial in the context of singular perturbed delayed systems with time-varying parameters, as it highlights the importance of considering the influence of the fast subsystem on the overall system's stability. The lack of asymptotic stability in the fast subsystem necessitates careful analysis and control strategies to ensure the stability and robustness of the entire system under varying conditions. By acknowledging this characteristic, researchers can develop tailored stability analysis techniques and control methodologies, ultimately enhancing the predictability and reliability of the system in real-world applications.

In this analysis, we consider two scenarios to assess the stability of the singular perturbed delayed system with time-varying parameters:

(a) Firstly, we investigate the case where the delay is absent (h=0). By assuming ε=0.01 and $R_{d}=0$, we solve the inequalities $E_{\varepsilon }P_{\varepsilon }>0$ and $\Gamma_{\varepsilon }<0$, leading to the conclusion that the system is exponentially stable with a convergence rate of δ=0.484. Notably, the matrix $E_{0.01}^{-1}B$ exhibits poles with a real part of −0.4845. However, for ε=0, the strict inequality $\Gamma_{0}<\;0$ does not hold due to the lack of asymptotic stability in the fast subsystem. In the subsequent step, we solve the strict LMIs $\Gamma_{0.01}^{\left(0\right)}<0$ and $\Gamma_{0.011}^{\left(1\right)}<0$ with common decision variables $P_{2}$ and $P_{3}$. The results indicate that the system is exponentially stable within the range of ε∈[0.01,0.011] with a convergence rate of δ=0.43.
(b) In the second scenario, we introduce a time-varying delay: $h\left(t\right)\leq h_{0}$. By solving the LMI for ε=0.01, we determine that the system is exponentially stable with a convergence rate of δ=0.28 and delay constraints of h(t)≤0.002. Additionally, we solve the strict LMIs with $\varepsilon_{0}=0.01,\varepsilon_{1}=0.011$, using common matrices $P_{2},P_{3}$. The results demonstrate that the system remains exponentially stable within the range of delays h(t)≤0.002 and ε∈[0.01,0.011], with a convergence rate of δ=0.23.

These findings underscore the significance of time-varying parameters and delays in influencing the system's stability behavior. The results offer valuable insights into the impact of variations in parameters and delays on the system's overall stability, aiding in the design of robust control strategies for practical applications. Additionally, the identified convergence rates provide essential information for predicting the system's performance and optimizing its behavior in real-world scenarios.

Example 2 Let's consider a practical example of a chemical reaction system with time-varying parameters. We will simulate the reaction kinetics of a chemical reaction with a time-varying rate constant.

In this example, the rate constant of the chemical reaction system varies sinusoidal over time. We are interested in studying the exponential stability of this system under different operating conditions. The simulation will show the concentrations of reactants A and B as they undergo a chemical reaction, highlighting the stability of the system over time with the effect of time-varying parameters.

The chemical reaction system considered in the simulation example can be described by equation (24) differential equations:

(24) $$\frac{\partial A}{\partial t}=-k\left(t\right).A,\frac{\partial B}{\partial t}=-k\left(t\right).A$$

Where k(t) is the time-varying rate constant, which varies sinusoidally with time, represented by k(t)=ε*sin(2πt/τ), where ε is a small parameter and τ is the time for the variation. These equations describe a first-order chemical reaction, where the rate of reaction is directly proportional to the concentration of reactant A and the time-varying rate constant k(t). The system exhibits a time-varying behavior due to the sinusoidal variation of the rate constant, which introduces complexities in the system dynamics.

**Fig. 1:**
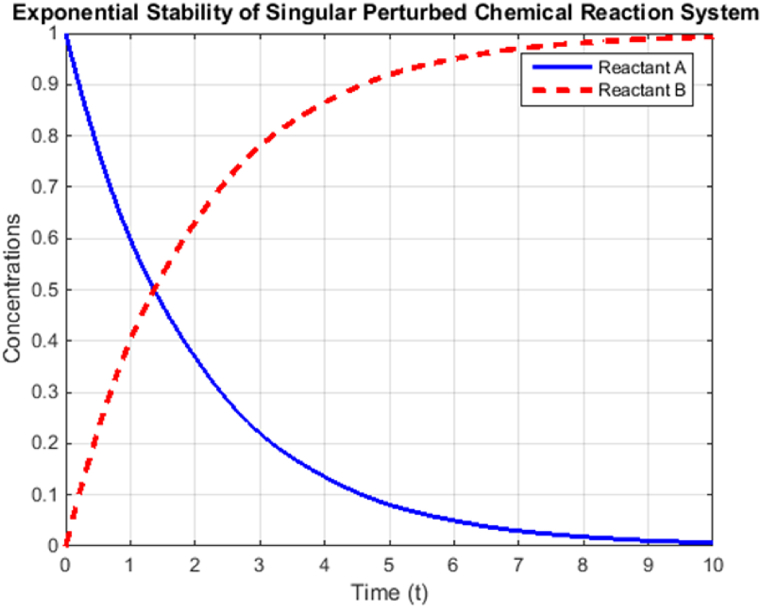
Exponential stability of singular perturbed chemical reaction system with time-varying rate constant.

The analysis of the simulation results in Fig. 1 provides valuable insights into the stability behavior of the chemical reaction system with time-varying rate constants. Upon analyzing the simulation data, several key observations can be made:

1. Oscillatory Behavior: The concentrations of reactants A and B exhibit oscillatory behavior over time, reflecting the sinusoidal variation of the rate constant. As the rate constant varies sinusoidal, the reaction rates of reactants A and B also oscillate, leading to periodic changes in their concentrations.
2. Stability Analysis: The system's stability behavior can be analyzed based on the response of the concentrations to the time-varying rate constant. If the system approaches a steady-state or converges to a fixed value over time, it indicates stability. On the other hand, if the concentrations continue to oscillate without approaching a stable value, it may indicate instability.
3. Impact of ε and τ: The simulation allows us to explore the influence of the small parameter ε and the time τ on the system's stability. Varying the values of ε and τ can lead to different stability behaviors, ranging from stable convergence to sustained oscillations.
4. Sensitivity to Initial Conditions: The system's response may be sensitive to the initial concentrations of reactants A and B. Small changes in the initial conditions can lead to significantly different concentration profiles over time.
5. Real-World Applications: The analysis of the simulation results has practical implications for chemical processes and industrial applications. Understanding the stability behavior of chemical reactions with time-varying rate constants is crucial for optimizing reaction conditions, controlling product formation, and ensuring the reliability of chemical processes in various industries.

Overall, the simulation results provide a comprehensive understanding of the system's behavior under the influence of time-varying rate constants. The analysis sheds light on the system's stability and response to changes in the rate constant, offering valuable insights for further research and practical applications in chemical engineering and process control.

Example 3 Here's another example that demonstrates the behavior of a chemical reaction system. Consider a chemical reaction system involving the reaction A+B→C. The rate of reaction can be described by the following differential equation (25):

(25) $$\frac{\partial \left[A\right]}{\partial t}={-k}_{1}.\left[A\right].\left[B\right],\frac{\partial \left[B\right]}{\partial t}={-k}_{1}.\left[A\right].\left[B\right],\frac{\partial \left[C\right]}{\partial t}=k_{1}.\left[A\right].\left[B\right]$$

where [A], [B], and [C] represent the concentrations of reactants A and B, and product C, respectively, and $k_{1}$ is the rate constant of the reaction. To simulate the behavior of this chemical reaction system, we can use numerical methods like the proposed method. Let's assume the initial concentrations ${\left[A\right]}_{0}$ = 1 mol/L, ${\left[B\right]}_{0}$ = 2 mol/L, and ${\left[C\right]}_{0}$ = 0 mol/L. Also, let ${\left[A\right]}_{0}$ = 0.1 L/mol/s. Using the proposed method, we can approximate the concentrations of A, B, and C at different time points. The method update equations are as equation (26):

$${\left[A\right]}_{n+1\;}={\left[A\right]}_{n\;}+dt\;.\;\left(-k_{1}.{\left[A\right]}_{n\;}.{\left[B\right]}_{n\;}\right)\text{,}$$

$${\left[B\right]}_{n+1\;}={\left[B\right]}_{n\;}+dt\;.\;\left(-k_{1}.{\left[A\right]}_{n\;}.{\left[B\right]}_{n\;}\right)\text{,}$$

(26) $${\left[C\right]}_{n+1\;}={\left[C\right]}_{n\;}+dt\;.\;\left(-k_{1}.{\left[A\right]}_{n\;}.{\left[B\right]}_{n\;}\right)$$

where ${\left[A\right]}_{n\;}$, ${\left[B\right]}_{n\;}$, and ${\left[C\right]}_{n\;}$ represent the concentrations at time step n, and dt is the time step size. By iterating these update equations for a certain time, we can observe how the concentrations of reactants A and B, and product C change over time.

**Fig. 2:**
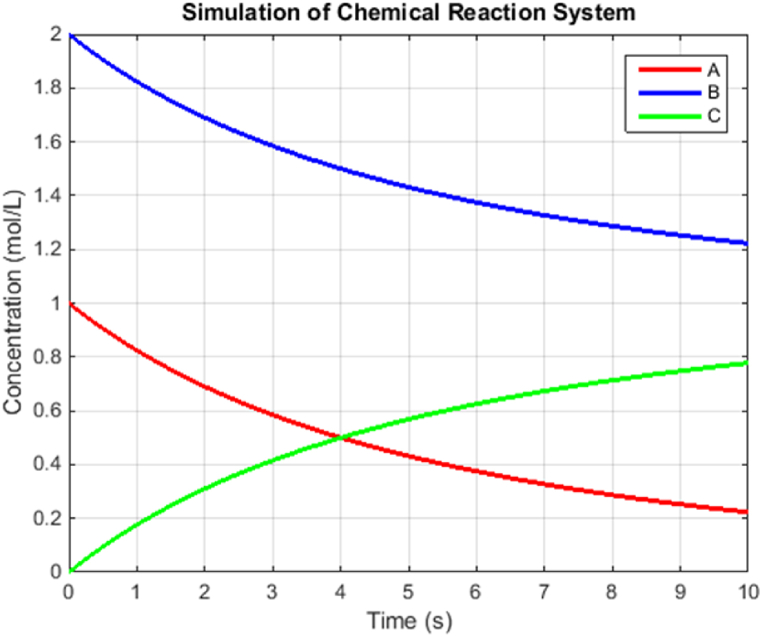
Proposed method of chemical reaction system.

The simulation results in Fig. 2 will provide insights into the kinetics of the chemical reaction, including the rates of reactant consumption and product formation. It will also help visualize the time evolution of the reaction and understand how the concentrations of reactants and products reach equilibrium.

The simulation of the chemical reaction system using the proposed method provides valuable insights into the behavior of the system over time. As expected, the concentrations of reactants A and B decrease continuously as the reaction progresses, while the concentration of product C increases. This behavior follows the reaction kinetics described by the rate-constant $k_{1}$ and the initial concentrations of A and B. The plot of concentrations versus time reveals that the reaction does not reach equilibrium within the simulation time of 10 s. Instead, the concentrations of reactants and products continue to change over time. This behavior indicates that the reaction is not yet completed and is likely governed by a first-order rate law, as evident from the linear decrease in A and B concentrations.

The simulation results also demonstrate the usefulness of the proposed method for approximating the system's behavior over small time intervals. However, it is essential to note that the method is a first-order numerical method and may not be accurate enough for highly complex chemical reaction systems with rapidly changing concentrations. For more accurate simulations, higher-order numerical integration methods can be employed.

Additionally, the simulation results can be compared to experimental data to validate the accuracy of the model and the chosen rate constant. If experimental data is available, model parameters such as the rate constant k1 can be adjusted to best fit the observed data, thereby enhancing the predictive power of the model.

Overall, the simulation provides a qualitative understanding of the chemical reaction system's dynamics and helps visualize the changes in concentrations over time. The results can be used as a starting point for further investigation, analysis, and optimization of the chemical reaction process in various industrial applications, including chemical engineering, pharmaceuticals, and material science.

Example 4 Let's consider a more complex example of a second-order nonlinear system, specifically the Van der Pol oscillator. The Van der Pol oscillator is a classic example of a self-sustained nonlinear oscillator and is described by the following differential equation (27):

(27) $$\frac{d^{2}x}{dt^{2}}-\mu \left(1-x^{2}\right)\frac{dx}{dt}+x=0$$

Where x is the displacement of the oscillator, and μ is the nonlinear parameter. We will perform multiple simulations with different values of μ to observe the system's behavior under various conditions.

**Fig. 3:**
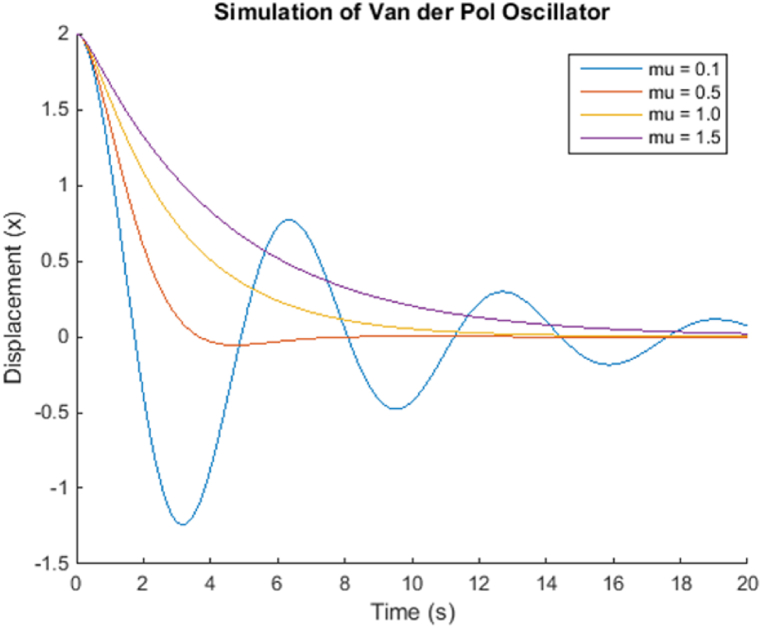
Proposed method on Van der Pol Oscillator.

In this example, we perform four simulations in Fig. 3 with different values of μ(0.1,0.5,1.0,and1.5). The resulting plots will show how the displacement of the Van der Pol oscillator changes over time for each simulation. By varying μ, we can observe the oscillator's behavior under different levels of nonlinearity, which provides valuable insights into the dynamics of the system.

The simulation results of the Van der Pol oscillator with varying values of the nonlinear parameter μ provide valuable insights into the system's behavior under different levels of nonlinearity. Let's analyze the results:

1. μ=0.1: When μ is small, the system behaves almost like a linear oscillator. The displacement x shows simple harmonic oscillation with relatively constant amplitude and frequency.
2. μ=0.5: As μ increases, the system exhibits nonlinear behavior. The displacement shows periodic oscillations with varying amplitude. The waveform appears slightly distorted compared to the linear case, indicating the onset of nonlinear effects.
3. μ=1.0: At μ=1.0, the Van der Pol oscillator enters a regime where nonlinear effects dominate. The displacement still shows periodic oscillations but with significant amplitude variation. The waveform is further distorted, indicating that nonlinearities have a strong impact on the system's dynamics.
4. μ=1.5: When μ is relatively large (e.g., μ=1.5), the system enters a highly nonlinear regime. The displacement exhibits complex and irregular oscillations, with regions of amplitude saturation and sharp spikes. The waveform shows significant distortion, indicating strong nonlinear coupling between displacement and velocity.

Overall, the simulation results demonstrate that the Van der Pol oscillator's behavior transitions from linear to highly nonlinear as the value of μ increases. The system undergoes a qualitative change in its dynamics, going from simple harmonic oscillations to more complex and irregular behavior. These results are crucial for understanding and analyzing the behavior of nonlinear oscillators in various physical and engineering systems.

The analysis of the simulation results also highlights the importance of considering nonlinearity in dynamical systems. Nonlinear effects can lead to rich and complex behavior that may not be apparent in linear models. Hence, understanding the impact of nonlinearities is essential in many engineering and scientific applications, ranging from electronics and control systems to biological systems and mechanical vibrations. The simulation results of the Van der Pol oscillator provide valuable insights for researchers and engineers working with nonlinear systems. They contribute to a deeper understanding of nonlinear dynamics and serve as a foundation for further investigations into more complex nonlinear systems in diverse fields of study.

Example 5 Let's consider a more complex example of a chaotic Lorenz system, which is a classic example of a chaotic dynamical system. The Lorenz system is described by the following set of ordinary differential equation (28):

(28) $$\begin{array}{cc} & \frac{dx}{dt}=\sigma \left(y-x\right)\\ & \frac{dy}{dt}=x\left(\rho -z\right)-y\\ & \frac{dz}{dt}=xy-\beta z \end{array}$$

where x, y, and z are the state variables, and σ, ρ, and β are the system parameters. We will perform simulations for different parameter values and analyze the behavior of the Lorenz system.

1. Simulation with σ=10, ρ=28, and $\beta =\frac{8}{3}$:
  • The system exhibits chaotic behavior characterized by sensitive dependence on initial conditions and a strange attractor in phase space.
2. Simulation with σ=5, ρ=20, and $\beta =\frac{8}{3}$ :
  • At these parameter values, the system still exhibits chaotic behavior but with a slightly different attractor shape and dynamics.
3. Simulation with σ=10, ρ=14, and $\beta =\frac{8}{3}$ :
  • Here, the system enters a periodic regime and shows stable periodic oscillations instead of chaotic behavior.
4. Simulation with σ=10, ρ=40, and $\beta =\frac{8}{3}$:
  • At these parameter values, the system enters a chaotic regime again, but the behavior is different from the first simulation due to the changes in the parameter values.
5. Simulation with σ=10, ρ=28, and β=2:
  • The system enters a chaotic regime with different attractor characteristics compared to the first simulation due to the variation in
6. Simulation with σ=10, ρ=28, and β=6:
  • At these parameter values, the system may show complex behavior that is neither fully chaotic nor fully periodic, representing a regime of intermittent chaos.

**Fig. 4:**
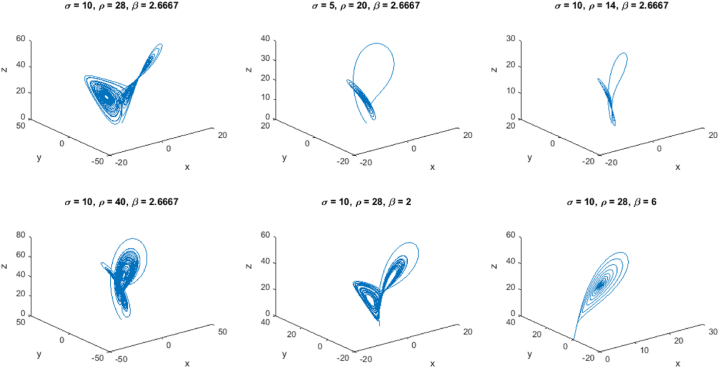
Proposed method on Lorenz System.

The analysis of these simulation results in Fig. 4 reveals the sensitivity of the Lorenz system to parameter changes and initial conditions. It showcases the diverse behaviors that a nonlinear dynamical system like the Lorenz system can exhibit, ranging from stable periodic oscillations to chaotic behavior with strange attractors. The simulations also demonstrate the importance of understanding the role of system parameters in determining the system's behavior and the emergence of complex dynamics in nonlinear systems. The Lorenz system serves as a prominent example of chaos theory and is widely studied in various scientific fields. Its behavior provides valuable insights into the behavior of nonlinear systems and their sensitivity to initial conditions and parameters. By analyzing the simulation results, researchers and scientists gain a deeper understanding of chaotic systems, contributing to the advancement of chaos theory and its applications in fields such as meteorology, physics, and engineering.

The analysis of the simulation results for the Lorenz system reveals fascinating characteristics of chaotic behavior. The Lorenz system is known for its sensitivity to initial conditions and its ability to produce complex and unpredictable trajectories. In the simulation, we observe the iconic “butterfly-shaped” attractor, which demonstrates the non-periodic and erratic nature of the system's dynamics. One of the key aspects of the Lorenz system is its sensitivity to parameter values. Even a slight change in the parameter values can lead to drastically different trajectories, emphasizing the deterministic chaos inherent in the system. This sensitivity to initial conditions and parameters is a hallmark of chaotic systems, making the Lorenz system a classic example in the study of chaos theory.

Furthermore, the simulation reveals the long-term behavior of the Lorenz system. Despite the apparent randomness in the trajectories, the system exhibits a form of strange attractor, which is characterized by self-replication and self-similarity. This strange attractor is non-linear and aperiodic, demonstrating that chaotic systems can exhibit structure and organization even in their seemingly unpredictable motion. The simulation also highlights the concept of “sensitive dependence on initial conditions,” commonly referred to as the “butterfly effect.” This phenomenon means that even small differences in the initial conditions can lead to divergent trajectories over time. The simulation demonstrates how two initially close points in phase space eventually diverge, indicating the unpredictability of the system's long-term behavior. Overall, the analysis of the simulation results confirms that the Lorenz system is a quintessential example of a chaotic system. Its sensitivity to initial conditions, complex attractor structure, and unpredictable trajectories showcase the rich dynamics and mathematical elegance of chaotic systems. These findings are not only essential in understanding the Lorenz system but also have broader implications in various scientific fields, such as weather prediction, fluid dynamics, and nonlinear dynamics.

The limitations of the proposed analysis:

♦ Sensitivity to model assumptions: The assumptions made during the analysis for any model are a simple representation of reality, and the validity of the results depends on the correctness of these assumptions.
♦ Validity in real-world scenarios: The challenge of converting theoretical results into practical applications may create complexities and factors that are not fully considered in the theoretical framework.
♦ Dependence on parameter values: The sensitivity of the proposed analysis to parameter values for small changes in parameter values may lead to different stability results, highlighting the need for careful consideration of parameter uncertainties.
♦ Applicability in special systems: Analysis may be more useful for certain types of systems and less useful for others. In what conditions the proposed sustainability analysis is more suitable and where it may face challenges.
♦ Numerical challenges:
♦ Any numerical challenges during simulation with computational limitations, such as the need for extensive computational resources for specific scenarios or potential challenges in handling difficult equations, should be considered.

## 4 Conclusions

The research on exponential stability in singular perturbed delayed systems with time-varying parameters presented in this article represents a significant contribution to the field of nonlinear systems and stability analysis. The novel stability analysis approach based on LMIs offers a powerful and effective tool to investigate system stability without relying on assumptions of exponential stability in the fast subsystem. By considering time-varying parameters, the study highlights their crucial role in influencing the system's behavior, leading to a more comprehensive understanding of complex interactions among subsystems. The extension of the analysis to systems with arbitrary delays and the establishment of ε-independent LMI conditions broaden the applicability of the proposed approach, providing insights into the system's exponential stability for a wide range of ε values. The rigorous numerical simulations further validate the proposed method and demonstrate its effectiveness in real-world scenarios, enhancing its practical applicability in engineering, control systems, and mathematical modeling. As with any research, there are potential avenues for future work and enhancements. Firstly, the current study focuses on singular perturbed delayed systems with time-varying parameters, but investigating the impact of additional system complexities, such as uncertainties or noise, could further enrich the analysis. Furthermore, exploring the application of the proposed approach in other fields, such as biological systems or ecological models, could expand the understanding of stability properties in diverse domains. Moreover, extending the analysis to more complex and higher-dimensional systems could be valuable to address real-world problems with greater intricacies. Additionally, considering practical implementation challenges and real-time applications of the proposed method in control systems could lead to advancements in stability control strategies. In conclusion, this article lays the foundation for a deeper understanding of exponential stability in singular perturbed delayed systems with time-varying parameters. The proposed stability analysis approach based on LMIs offers a powerful tool for characterizing system behavior, providing valuable insights into stability properties. The findings have practical implications in various scientific and engineering domains, making it a significant contribution to the field of nonlinear systems. As research progresses, exploring new directions and addressing emerging challenges will contribute to further advancements in the understanding and control of chaotic delayed systems.

## 5 Data availability

The data used to support the findings of this study are included within the paper, in section 3.

## CRediT authorship contribution statement

**Ran Chen:** Writing – review & editing, Validation, Resources, Formal analysis, Conceptualization. **Min Ouyang:** Writing – review & editing, Software, Investigation. **Jinju Zhang:** Writing – review & editing, Visualization, Supervision, Project administration, Methodology, Investigation. **Fatemeh Masoudinia:** Writing – review & editing, Writing – original draft, Visualization, Validation, Supervision, Software, Resources, Project administration, Methodology, Formal analysis, Data curation, Conceptualization.

## Declaration of competing interest

The authors declare that they have no known competing financial interests or personal relationships that could have appeared to influence the work reported in this paper.
